# Impact of the COVID-19 pandemic on the pharmacological, physical, and psychological treatments of pain: findings from the Chronic Pain & COVID-19 Pan-Canadian Study

**DOI:** 10.1097/PR9.0000000000000891

**Published:** 2021-02-08

**Authors:** Anaïs Lacasse, M. Gabrielle Pagé, Lise Dassieu, Nadia Sourial, Audrée Janelle-Montcalm, Marc Dorais, Hermine Lore Nguena Nguefack, Marimée Godbout-Parent, Maria Hudspith, Gregg Moor, Kathryn Sutton, James M. Thompson, Manon Choinière

**Affiliations:** a Département des Sciences de la Santé, Université du Québec en Abitibi-Témiscamingue (UQAT), Rouyn-Noranda, QC, Canada; b Centre de recherche du Centre hospitalier de l'Université de Montréal (CRCHUM), Montréal, QC, Canada; c Département d'anesthésiologie et de médecine de la douleur, Faculté de médecine, Université de Montréal, Montréal, QC, Canada; d Département de médecine de famille et de médecine d'urgence, Faculté de médecine, Université de Montréal, Montréal, QC, Canada; e StatSciences Inc., Notre-Dame-de-l'Île-Perrot, QC, Canada; f Pain BC Society, Vancouver, BC, Canada; g Department of Public Health Sciences, Queens University, Kingston, ON, Canada; h Department of Family Medicine, Dalhousie University, Halifax, NS, Canada

**Keywords:** Chronic pain, COVID-19, Pain management, Web-based cross-sectional study

## Abstract

**Introduction:**

Multimodal treatment is recognized as the optimal paradigm for the management of chronic pain (CP). Careful balance between pharmacological and physical/psychological approaches is thus desirable but can be easily disrupted.

**Objectives:**

This study aimed at exploring the impact of the COVID-19 pandemic on pharmacological and physical/psychological treatments of CP.

**Methods:**

A Pan-Canadian cross-sectional web-based study was conducted between April 16th and May 31st 2020 among adults living with CP when the country was in the ascending slope of the first COVID-19 pandemic wave.

**Results:**

A total of 2864 participants shared their treatment experience (mean age: 49.7 years and women: 83.5%). Among medication users (n = 2533), 38.3% reported changes in their pharmacological pain treatment. The main reasons were as follows: (1) changes in pain symptoms, (2) lack of access to prescribers/cancellation of medical appointments, and (3) increased medication intake in compensation for stopping physical/psychological treatments because of the pandemic. Among participants who used physical/psychological pain management approaches before the pandemic (n = 2467), 68.3% had to modify their treatments or self-management strategies. Common reasons were lack of access to clinics/exercise facilities and the need to compensate for having to stop another type of physical/psychological treatment because of the pandemic-related public health safety measures.

**Conclusions:**

Our study underlines the negative impact of the COVID-19 pandemic on access to pain relief, which is considered a fundamental human right. Results will help to justify resource allocation and inform the development of interventions to be better prepared for waves to come and future health crises.

## Abstract 2

The COVID-19 pandemic had, and probably continues having, a significant negative impact on access to pharmacological, physical, and psychological pain treatments.

## 1. Introduction

The first Canadian case of COVID-19 was reported in January 2020.^8^ Community transmission was confirmed in March 2020, and rapid closure of daycares, schools, workplaces, and nonessential services occurred to varying degrees across the Canadian provinces and territories.^7,32^ Restrictions to private and public gatherings in addition to closure of regional/provincial/national/international borders were also put in place. The biopsychosocial effects of the COVID-19 pandemic are important in the general population^50,54^ and affect disproportionately high-risk and vulnerable individuals such as those living with chronic diseases, older adults, and socially and economically deprived populations.^9,52^ Public health restrictions and pandemic-related stress thus added to the preexisting physical, psychosocial, and financial burden that is known to be associated with chronic pain (CP).^14,66^ Moreover, persistent users of prescribed opioids have been identified as a vulnerable group in the COVID-19 pandemic because the context makes it difficult to adhere to all prescribing and follow-up opioid guidelines.^39^ Opioids have also been shown to weaken the immune system.^57^

Multimodal treatment is recognized as the optimal paradigm for the management of CP.^10^ A careful balance between pharmacological and physical/psychological approaches is therefore desirable, but it can be hard to achieve and easily disrupted.^3,12^ Since the beginning of the pandemic, many challenges led us to believe that the pharmacological treatment of CP would be substantially impacted. Case in point, uncertainty regarding the use of nonsteroidal anti-inflammatory drugs during the pandemic^29,59^ was broadcast throughout the media in March 2020, which might have sparked concerns among patients and prescribers. Also, in some health care jurisdictions, patients living with certain arthritic conditions (eg, rheumatoid arthritis, erosive osteoarthritis of the hands) were denied hydroxychloroquine/chloroquine treatment in an effort to prevent a shortage of these drugs^31^ because these antimalarial were initially examined as a treatment for COVID-19.^17,53^ Shortages of opioids were also announced by some Canadian Health Technology Assessment agencies in early June 2020.^30^ The demand for dexamethasone also peaked when it was suggested to be an effective treatment for COVID-19 in June 2020.^41^ Furthermore, many multidisciplinary pain treatment clinics were closed or reduced their services.^14,40^ The confinement measures along with personal financial losses may also have limited access to many other types of treatments (eg, physical therapy, psychological counselling, and self-help groups).^14,33^ Finally, fear of going to health care appointments^14^ and nonmedical use of prescribed medications or illicit drugs^20^ were expected. Although major disruptions in CP management were anticipated by the research and the clinical communities,^14,16,19,23,24,33,35,40,53,57,66^ the impact of the pandemic among individuals living with CP still needed to be quantified.

This study aimed at documenting the impact of the COVID-19 pandemic on the pharmacological and physical/psychological management of CP. Participants' characteristics associated with changes in pain treatments during the pandemic were also examined.

## 2. Methods

### 2.1. Study design and sample

This study was part of a larger initiative, the Chronic Pain & COVID-19 Pan-Canadian Study,^51^ which used a web-based, cross-sectional, and mixed-method design to answer various research questions surrounding how CP patients experienced the pandemic. Individuals aged 18 years and older, living in Canada, reporting pain for more than 3 months,^63^ and able to complete a web-based self-administered questionnaire in French/English were eligible. The study was approved by the Centre hospitalier de l'Université de Montréal's Research Ethics Board. Patient partners were involved in every step of the study.

### 2.2. Web-based data collection

Data were collected with SurveyMonkey. A web-based recruitment strategy, which was shown as effective in previous studies,^37,38^ was implemented to reach a community sample of Canadian living with CP. The questionnaire's hyperlink that led potential participants to the landing page of the study where enough information was provided for informed consent was included in the invitation message.

The recruitment strategy included the following: (1) advertisement by national and provincial CP patient associations through their e-newsletter, social media page, and/or website, (2) invitations shared among various Facebook support groups related to CP or associated conditions, (3) email invitations and social media posts (Facebook and Twitter) shared by colleagues and friends from various socioeconomic statuses (snowballing technique), and (4) email invitations and social media posts shared by local, provincial, and national research networks. The study was also announced on the intranets and in press releases issued by the principal investigators' institutions and was subsequently covered in various broadcasts and text interviews published on the web. To maximize study participation, individuals who completed the questionnaire were offered the possibility to enter a draw to win 1 of 10 $100 prepaid gift cards. Multiple participations from the same person were minimized by the survey platform. The questionnaire was pretested by 5 French-speaking and English-speaking individuals living with CP with various levels of education.

The study was open from April 16 to May 31, 2020, roughly 1 month after a state of emergency was declared in most Canadian provinces; the first province to declare a state of emergency was Quebec on March 12th, and the last one was Nova Scotia on March 22nd.^7^ The questionnaire was available when the cumulative cases of the first COVID-19 pandemic wave were growing exponentially in some provinces (ie, in Quebec, Ontario, and Alberta) and during the peak of daily reported new cases in Canada.^26^ The recruitment was stopped when announcements for some loosening up of the public health safety restrictions were being made.

### 2.3. Study Variables

Changes in pharmacologic pain treatments were assessed using a close-ended question (“did you modify your pharmacological treatments for pain because of the COVID-19 pandemic? [eg, opioids, anti-inflammatories, or any other prescribed or over-the-counter medications]”), followed by the answers “yes,” “no,” and “not applicable because I do not take any pain medications.” A similar question was presented for physical/psychological treatments. Participants who answered “yes” were asked to explain why and how they had modified their treatments using an open-ended question. This question format was chosen because of the exploratory nature of this study and the aim of collecting more insights and a wider range of responses. The reasons collected (verbatim) were reviewed individually to develop a standardized coding system. Coding was then achieved independently by 2 authors who reached consensus for any nonconcordance afterwards. Other variables covered in the web-based questionnaire are presented in Table 1.

**Table 1:** Covariables measured in the web-based questionnaire.

Pain characteristics	Items covered pain duration, pain frequency, body location (including presence of generalized pain or multisite pain defined as ≥2 body sites), intensity (0–10 numerical rating scales for average pain intensity in the past 7 d and when pain was at its worst in the past 7 days^21,34^), and unpleasantness (0–10 numerical rating scale for average pain unpleasantness in the past 7 days^21^). Pain interference in the past 7 days was assessed with the Brief Pain Inventory (BPI).^15^ Participants were also asked information about their primary pain care provider.
COVID-related variables	Changes in pain symptoms since the beginning of the pandemic was assessed using a 7-point Patient Global Impression of Change (PGIC)^21^ scale. The questionnaire also assessed if participants had needed to renew their pain medication prescriptions since the beginning of the pandemic, if they had a confirmed or pending diagnosis of COVID-19 infection, current COVID-like symptoms, any contact with a person with confirmed or pending diagnosis in the past 30 days, if they had travelled outside Canada since January 2020, had their employment status changed during the pandemic, and if they consulted a specialized COVID-19 screening clinic in the past 2 weeks. Finally, they reported their level of stress in relation to the pandemic on the question “on a scale from 0 (not at all) to 10 (extremely), to what extent do you find the COVID-19 pandemic stressful?”
General health	Psychological distress symptoms in the past month was assessed with the anxiety/depression subscales of the Patient Health Questionnaire (PHQ-4),^36^ which allows for the classification of the total scores into severity groups. Participants were also asked if a health care provider had told them that their immune system was weak or compromised. Self-perceived health was assessed with the EQ-5D-5L 0–100 item^28^ ranging from “worst health you can imagine” to “best health you can imagine.”
Sociodemographic profile	Age, sex (female, male, and undetermined), race/ethnicity (statistics Canada's categories^60^), education, employment status before public health measures were first deployed in their province, current living arrangement, marital status, province of residence, and the first 3 characters of their postal code (allowing for an urban vs rural classification). The dates of the questionnaire completion were used to classify participants within 7 categories representing the 7 calendar weeks during which the questionnaire was available.

### 2.4. Data analysis

Our study sample included the participants who completed the items about changes in pain treatments. Scores on pain characteristics which were measured at the very beginning of the questionnaire and which had very few missing data points were compared between participants who completed the items about changes in pain treatments vs those with missing data to detect any clinically significant differences (>20% differences^22^). Descriptive statistics were calculated to summarize the participants' characteristics, changes in pain treatments, and reasons behind the changes. Proportions of participants reporting changes in their pharmacological or physical/psychological treatments were stratified according to factors of interest such as the week during which the questionnaire was completed, sex, province of residence, and body location of pain symptoms.

Among users of pain medications, a multivariable logistic regression model was used to identify participants' characteristics associated with changes in pharmacologic treatments during the pandemic (dependent variable). A similar model was achieved to identify participants' characteristics associated with changes in physical/psychological pain treatments. A broad set of a priori determined variables potentially associated with such changes were included in the multivariable logistic regression models. Choice of variables was based on the existing scientific literature and/or clinical plausibility, as well as on the predisposing, enabling, and need factors proposed in Andersen's model (1995),^1^ a widely used model in health care utilization studies.^2^ Variance inflation factors were used to help detect and remove variables with multicollinearity (variance inflation factors < 5^65^).

## 3. Results

Between April 16 and May 31, 2020, a total of 3428 individuals from all over Canada accessed the web-based questionnaire and 3159 participants living with CP ultimately met inclusion criteria (167 individuals reporting pain for less than 3 months and 102 with missing data regarding pain duration were excluded). Our sample was composed of the 2864 participants who shared their treatment experience. No clinically significant differences were found between those who answered the questions about changes in their treatments vs those who did not in terms of pain duration (47.2% vs 42.2% reporting pain for >10 years), pain frequency (92.3% vs 81.9% experiencing pain continuously), and presence of generalized pain (34.6% vs 26.4%). However, a greater proportion of participants included in this study reported multisite pain (92.9% vs 65.1%).

Characteristics of the study participants are presented in Table 2. Age ranged from 18 to 99 years (mean: 49.7 ± 13.7), and 83.5% were women. Undetermined sex was reported by 11 participants (0.41%). A large majority reported using pharmacological pain treatments (88.4%) and physical/psychological pain treatments (87.3%). Only 24 participants (0.9%) had been diagnosed with COVID-19 at the time of questionnaire completion.

**Table 2:** Sociodemographic and clinical characteristics of the participants.

Characteristics (n = 2864)	n (%)*
Age (y), mean ± SD	49.74 ± 13.69
Sex	
Females	2218 (83.51)
Males	427 (16.08)
Undetermined	11 (0.41)
Race/ethnicity	
White	2346 (88.33)
Others	310 (11.67)
Postsecondary education	
Yes	2164 (82.22)
No	468 (17.78)
Employed full-time or part-time before public health measures were first deployed in their province	
Yes	976 (34.99)
No	1813 (65.01)
Province of residence	
Quebec	1221 (46.66)
Ontario	622 (23.77)
British Columbia	560 (21.40)
Alberta	76 (2.90)
Nova Scotia	41 (1.57)
New Brunswick	25 (0.96)
Manitoba	24 (0.92)
Saskatchewan	22 (0.84)
Newfoundland and Labrador	18 (0.69)
Prince Edward Island	7 (0.27)
Yukon	1 (0.04)
Northwest Territories/Nunavut	0 (0.00)
Pain duration	
≤2 y	354 (12.36)
3–5 y	509 (17.77)
5–10 y	649 (22.66)
>10 y	1352 (47.21)
Experience pain continuously	
Yes	2638 (92.30)
No	220 (7.70)
Pain intensity on average in the past 7 days (0–10)—mean ± SD	6.13 ± 1.84
Use of pharmacological pain treatments	
Yes	2533 (88.44)
No	331 (11.56)
Use of physical/psychological pain treatments	
Yes	2467 (87.27)
No	360 (12.73)
Change in pain symptoms since the beginning of the pandemic	
Considerably worsened/worsened a lot/somewhat worsened	1974 (69.02)
Unchanged	742 (25.94)
Somewhat improved/improved a lot/considerably improved	144 (5.03)
Needed to renew pain medication since the beginning of the pandemic	
Yes	1621 (64.27)
No	901 (35.73)
Diagnosis of COVID-19 confirmed by a test	
Yes	24 (0.85)
No	2797 (99.15)

* Unless stated otherwise.

**Figure 1.**
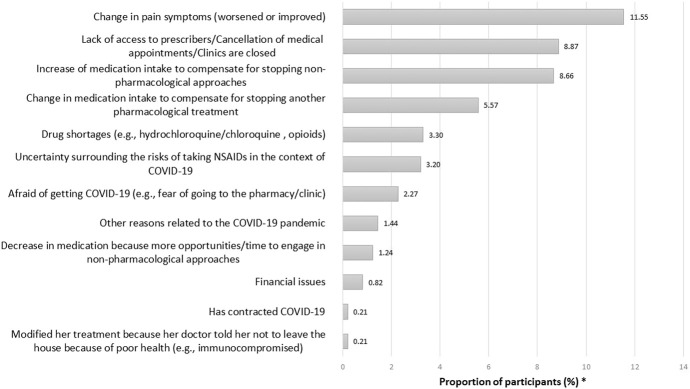
COVID-related reasons why participants reported having changed their pharmacological pain treatments during the pandemic. *Categories are not mutually exclusive because participants could list various reasons; N.B. 577 of the 970 participants who reported changes in their pain medication (59.48%) did not provide any specific reason (2.37%) or reasons not related to the COVID-19 pandemic (57.11%) –e.g., drug side effects and litigation with insurance company (data not shown in the graph).

**Figure 2.**
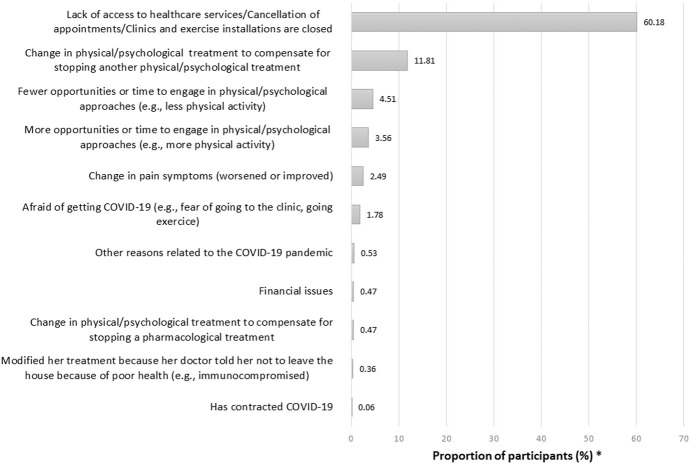
COVID-related reasons why participants reported having changed their physical/psychological pain treatments during the pandemic. *Categories are not mutually exclusive since participants could list various reasons; N.B. 277 of the 1685 participants who reported changes in their physical/psychological pain treatments (16.44%) did not provide any specific reason (0.83%) or reasons not related to the COVID-19 pandemic (15.61%) (data not shown in the graph).

**Figure 3.**
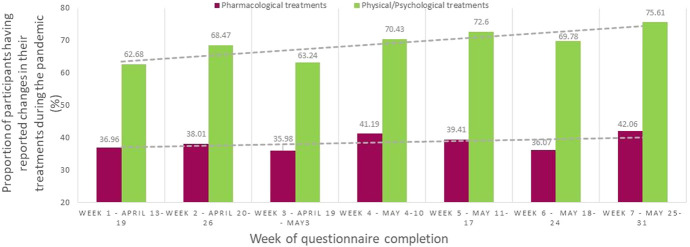
Proportions of participants having reported changes in their pharmacological or physical/psychological pain treatments during the pandemic according to the week of questionnaire completion.

**Figure 4.**
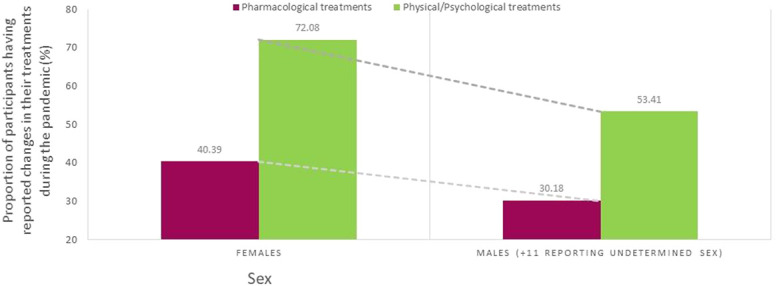
Proportions of participants having reported changes in their pharmacological or physical/psychological pain treatments during the pandemic according to sex.

**Figure 5.**
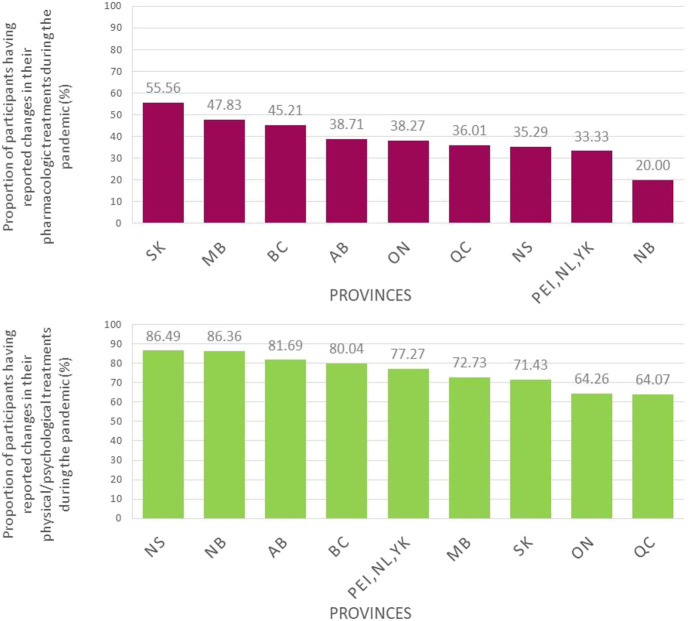
Proportions of participants having reported changes in their pharmacological (upper panel) or physical/psychological treatments (lower panel) during the pandemic according to province of residence. QC: Quebec, BC: British Columbia, AB: Alberta, SK: Saskatchewan, MB: Manitoba, ON: Ontario, NB: New Brunswick, NS: Nova Scotia, PEI: Prince Edward Island, NL: Newfoundland and Labrador, YK: Yukon; PEI (n = 7), NL (n = 18), and YK (n = 1) were grouped because of the small sample size.

**Figure 6.**
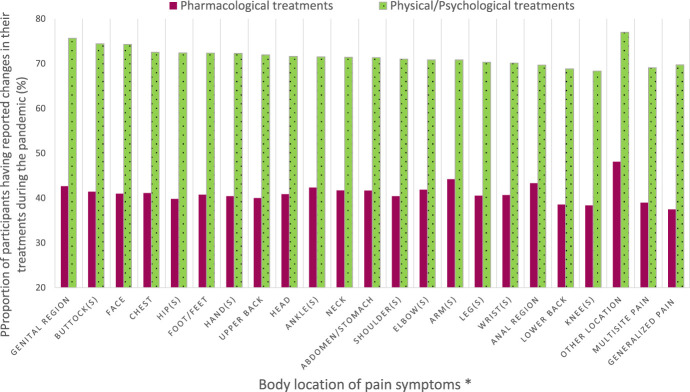
Proportions of participants having reported changes in their pharmacological or physical/psychological pain treatments during the pandemic according to the body location of pain symptoms. *Categories are not mutually exclusive since participants could list various body locations.

In the subsample of users of prescribed or over-the-counter pain medications (n = 2533), 38.3% reported changes in their pharmacological treatments during the pandemic. Among those participants, 40.5% reported COVID-related reasons, 57.11% non–COVID-related reasons (even if participants were asked explicitly about modifications made to their treatments because of the pandemic), and 2.4% no specific reason. COVID-related reasons for changes in pharmacological pain treatments during the pandemic are presented in Figure 1. The most common reasons were as follows: (1) change in pain symptoms because of the pandemic, (2) lack of access to prescribers, including cancellation of medical appointments or closed clinics, and (3) increase in medication intake in compensation for the stop of physical/psychological approaches. Among participants who used physical/psychological pain management approaches (n = 2467), 68.3% reported that they modified their treatments or self-management strategies during the pandemic. In this subsample, 83.6% reported COVID-related reasons, 15.6% non–COVID-related reasons, and 0.8% no specific reason. Common COVID-related reasons reported by participants (Figure 2) were lack of access to clinics/exercise installations or the need to change in compensation for stopping another type of physical/psychological treatments because of the pandemic. Few participants reported fear of going out or financial issues as reasons why changes were brought to their pharmacological or physical/psychological treatments. When looking at the week of questionnaire completion, the proportion of participants reporting changes in their pharmacological treatments was stable in time whereas changes in physical/psychological treatments appeared to be more common later during the pandemic (Figure 3). More women than men reported changes in their treatments (Figure 4). The percentage of participants reporting having modified their treatments seemed to vary substantially according to the province of residence (Figure 5), but not according to the body location of pain symptoms (Figure 6).

Results of the multivariable model used to identify participants' characteristics associated with changes in pharmacological pain treatments during the pandemic are presented in Table 3. The model included 61 dummy variables. Independent of other characteristics included in the model, 9 factors were found to be associated with an increased likelihood of pharmacological treatment modification: (1) change in pain symptoms since the beginning of the pandemic (OR worsened vs unchanged: 3.84, 95% confidence interval [CI]: 2.92–5.06; OR improved vs unchanged: 2.76, 95% CI: 1.65–4.62), (2) having needed to renew pain medication during the pandemic (OR: 1.79, 95% CI: 1.45–2.22), (3) not being followed in a family medicine group (FMG) for pain treatment (OR private clinic vs FMG: 1.91, 95% CI: 1.18–3.09; OR not receiving pain care vs FMG: 1.95, 95% CI: 1.31–2.90; OR other pain treatment vs FMG group: 1.39, 95% CI: 1.07–1.81; no difference regarding pain clinic vs FMG), (4) employment status change during the pandemic (OR miscellaneous status change vs continue to work and have to commute to work: 2.08, 95% CI: 1.30–3.34), (5) change in physical/psychological treatments during the pandemic (OR: 2.18, 95% CI: 1.71–2.78), (6) presence of psychological distress symptoms in the past month (OR severe vs none: 1.75, 95% CI: 1.21–2.54), (7) having a postsecondary education (OR: 1.36, 95% CI: 1.04–1.78), (8) being employed before the pandemic (OR: 1.56, 95% CI: 1.06–2.28), and (9) province of residence (OR British Columbia vs Quebec: 1.42, 95% CI: 1.02–1.96). Variables associated with a decreased likelihood of a change in pharmacological pain treatments during the pandemic were as follows: (1) reporting generalized pain (OR: 0.73, 95% CI: 0.58–0.90) and (2) week of questionnaire completion (OR week 6 vs week 1: 0.55, 95% CI: 0.33–0.90).

**Table 3:** Results of the multivariable logistic regression models used to identify participants' characteristics associated with changes in pain treatments during the pandemic.

Treatment users' characteristics*	Adjusted results regarding factors associated with changes in pharmacological pain treatments			Adjusted results regarding factors associated with changes in physical/pharmacological pain treatments		
	OR	95% confidence interval	*P*	OR	95% confidence interval	*P*
Pain characteristics						
Pain duration (vs ≤2 y)						
3–5 y	0.829	0.565–1.216	0.3363	0.973	0.631–1.501	0.9025
5–10 y	0.946	0.659–1.358	0.7622	0.899	0.597–1.353	0.6093
>10 y	0.995	0.706–1.401	0.9758	0.923	0.628–1.356	0.6829
Experience pain continuously (yes vs no)	0.848	0.556–1.293	0.4430	1.145	0.717–1.826	0.5710
Received a medical diagnosis for his/her pain (yes vs no)	0.877	0.628–1.224	0.4405	1.363	0.935–1.986	0.1075
Multisite pain (yes vs no)	1.238	0.776–1.976	0.3705	1.520	0.945–2.445	0.0842
Generalized pain (yes vs no)	**0.726**	**0.583**–**0.904**	**0.0042**	1.163	0.904–1.497	0.2400
Pain intensity on average in the past 7 days (0–10)	1.011	0.938–1.091	0.7674	**0.915**	**0.841–0.996**	**0.0393**
BPI interference score (0–10)	1.033	0.957–1.115	0.4027	1.041	0.957–1.132	0.3490
Primary pain care provider (vs family medicine group)						
Others	**1.393**	**1.074**–**1.805**	**0.0124**	1.303	0.967–1.755	0.0818
Private clinic	**1.912**	**1.183**–**3.090**	**0.0082**	0.848	0.502–1.432	0.5368
Pain clinic	1.174	0.913–1.511	0.2109	1.254	0.944–1.665	0.1181
Not receiving pain care	**1.949**	**1.309**–**2.902**	**0.0010**	0.702	0.450–1.097	0.1202
COVID-related variables						
Change in pain symptoms since the beginning of the pandemic (vs unchanged)						
Worsened	**3.843**	**2.919**–**5.059**	**<0.0001**	**2.452**	**1.898–3.169**	**<0.0001**
Improved	**2.757**	**1.646**–**4.620**	**0.0001**	**3.404**	**1.801–6.435**	**0.0002**
Needed to renew pain medication since the beginning of the pandemic (yes vs no)	**1.791**	**1.445**–**2.220**	**<0.0001**	1.067	0.843–1.351	0.5881
Diagnosis of COVID-19 confirmed by a test (yes vs no)	0.689	0.219–2.166	0.5233	0.759	0.222–2.592	0.6598
Waiting for a test or the results of a diagnostic test (yes vs no)	1.298	0.427–3.944	0.6457	0.541	0.164–1.780	0.3117
Currently having symptoms (cough and fever) that could be caused by COVID-19 (yes vs no)	1.166	0.661–2.056	0.5953	0.868	0.455–1.657	0.6682
Have been in physical contact or in proximity (less than 2 metres) with a person who has been diagnosed with COVID-10 or waiting for test results in the past 30 days (yes vs no)	0.878	0.505–1.529	0.6469	0.759	0.410–1.404	0.3795
Have travelled outside Canada since January 2020 (yes vs no)	1.067	0.802–1.419	0.6573	1.029	0.741–1.431	0.8629
Employment status during pandemic (vs continue to work and have to commute to work)						
Continued to work but remotely	1.202	0.790–1.829	0.3904	1.430	0.898–2.276	0.1319
Temporarily laid off	0.944	0.580–1.539	0.8185	1.651	0.969–2.814	0.0654
Permanently laid off	1.059	0.342–3.279	0.9213	1.061	0.361–3.118	0.9137
Temporary return to work to help during the crisis	0.286	0.030–2.750	0.2782	0.456	0.089–2.332	0.3453
No change/not working	1.636	0.980–2.732	0.0599	1.005	0.563–1.793	0.9860
Other/miscellaneous status changes	**2.082**	**1.300**–**3.335**	**0.0023**	1.447	0.839–2.495	0.1838
Consulted in a specialized screening clinic for COVID-19 over the past 2 weeks (yes vs no)	1.153	0.673–1.977	0.6040	0.976	0.522–1.823	0.9386
Change in physical/psychological pain treatments during the COVID-19 pandemic (change in pharmacological pain treatments during the COVID-19 pandemic for the second model)						
Yes (vs no)	**2.179**	**1.708**–**2.780**	**<0.0001**	**2.187**	**1.710–2.798**	**<0.0001**
Not applicable (vs no)	**2.114**	**1.482**–**3.016**	**<0.0001**	0.858	0.078–9.379	0.9000
Stress towards the pandemic (0–10)	0.998	0.951–1.047	0.9238	1.000	0.947–1.055	0.9921
General health						
Psychological distress symptoms according to the PHQ-4 (vs none)						
Mild	1.198	0.896–1.602	0.2220	**0.713**	**0.519–0.978**	**0.0359**
Moderate	1.369	0.987–1.901	0.0602	0.853	0.588–1.237	0.4023
Severe	**1.750**	**1.208**–**2.537**	**0.0031**	**0.601**	**0.394–0.915**	**0.0176**
Was told by a health care provider that his/her immune system was weak or compromised	1.017	0.826–1.252	0.8753	0.840	0.663–1.064	0.1486
Self-perceived health according to the EQ-5D-5L (0–100)	1.003	0.998–1.009	0.2300	1.000	0.994–1.006	0.9066
Sociodemographic profile						
Age (y)	0.997	0.988–1.006	0.5121	**0.980**	**0.970–0.990**	**0.0001**
Sex (females vs males/undetermined†)	1.177	0.882–1.570	0.2677	**1.752**	**1.304–2.354**	**0.0002**
Race/ethnicity (white vs others)	0.915	0.666–1.257	0.5838	1.359	0.954–1.936	0.0891
Postsecondary education (yes vs no)	**1.360**	**1.037**–**1.783**	**0.0262**	**2.093**	**1.578–2.775**	**<0.0001**
Employed full-time or part-time at the time public health measures were first deployed in their province (yes vs no)	**1.558**	**1.064**–**2.279**	**0.0226**	0.688	0.439–1.081	0.1045
Disabled at the time public health measures were first deployed in their province (yes vs no)	0.917	0.709–1.186	0.5095	1.128	0.847–1.501	0.4103
Living alone (yes vs no)	0.955	0.701–1.302	0.7719	1.330	0.946–1.871	0.1012
Marital status (vs married/common law)						
Single	0.893	0.667–1.197	0.4493	**0.562**	**0.406–0.779**	**0.0005**
Separated or divorced	1.182	0.848–1.648	0.3237	**0.541**	**0.374–0.781**	**0.0011**
Widowed	0.973	0.520–1.820	0.9308	0.607	0.312–1.182	0.1419
Province (vs Quebec‡)						
British Columbia	**1.415**	**1.024**–**1.955**	**0.0355**	**2.296**	**1.564–3.370**	**<0.0001**
Alberta	0.973	0.534–1.774	0.9282	**2.747**	**1.297–5.820**	**0.0083**
Saskatchewan	2.479	0.762–8.061	0.1312	1.628	0.439–6.038	0.4663
Manitoba	2.483	0.891–6.920	0.0819	1.143	0.370–3.533	0.8165
Ontario	1.173	0.875–1.572	0.2850	0.947	0.691–1.296	0.7326
New Brunswick	0.437	0.133–1.437	0.1727	3.155	0.819–12.147	0.0948
Nova Scotia	0.692	0.312–1.533	0.3643	2.655	0.952–7.409	0.0622
Prince Edward Island/Newfoundland and Labrador/Yukon	1.086	0.403–2.922	0.8706	2.036	0.620–6.688	0.2413
Residing in a rural area (vs urban)	1.193	0.902–1.578	0.2171	0.900	0.660–1.227	0.5050
Week of questionnaire completion (vs week 1)						
Week 2–April 20–26	0.833	0.597–1.164	0.2855	1.181	0.815–1.710	0.3796
Week 3–April 19–May 3	0.820	0.552–1.218	0.3255	1.119	0.725–1.727	0.6121
Week 4–May 4–10	0.752	0.488–1.159	0.1964	1.036	0.640–1.675	0.8863
Week 5–May 11–17	0.769	0.488–1.212	0.2572	1.144	0.686–1.907	0.6069
Week 6–May 18–24	**0.545**	**0.330**–**0.900**	**0.0177**	1.125	0.645–1.965	0.6776
Week 7–May 25–31	0.753	0.430–1.319	0.3210	1.279	0.661–2.477	0.4652

Bold text indicates statistically significant associations (95% confidence interval that excludes 1/*P*-value less than 0.05).

* 2135 and 1903 participants were included in the first and the second multivariate analysis, respectively.

† Too few participants reported underdetermined sex/gender identity (n = 11), so they were combined with men for the analysis.

‡ The province of Quebec was chosen as the reference category because it was the most severely affected in terms of number of COVID-19 cases and deaths.

BPI, Brief Pain Inventory; PHQ-4, Patient Health Questionnaire—4 items.

Results of the multivariable model used to identify participants' characteristics associated with changes in physical/psychological pain treatments are also presented in Table 3. Variables associated with an increased likelihood of physical/psychological treatment modification were as follows: (1) change in pain symptoms since the beginning of the pandemic (OR worsened vs unchanged: 2.45, 95% CI: 1.90–3.17; OR improved vs unchanged: 3.40, 95% CI: 1.80–6.44), (2) change in pharmacological treatments during the pandemic (OR: 2.19, 95% CI: 1.71–2.80), (3) being a female (OR: 1.75, 95% CI: 1.30–2.35), (4) having a postsecondary education (OR: 2.09, 95% CI: 1.58–2.78), and (5) province of residence (OR British Columbia vs Quebec: 2.30, 95% CI: 1.56–3.37; OR Alberta vs Quebec: OR: 2.75, 95% CI: 1.30–5.82). Factors associated with a decreased likelihood of a change in physical/psychological pain treatments during the pandemic were as follows: (1) higher average pain intensity in the past 7 days (OR: 0.92, 95% CI: 0.84–0.996), (2) presence of psychological distress symptoms in the past month (OR mild vs none: 0.713, 95% CI: 0.52–0.98; OR severe vs none: 0.60, 95% CI: 0.39–0.92), (3) older age (years) (OR: 0.98, 95% CI: 0.97 0.99), and (4) being single, separated, or divorced (OR single vs married/common law: 0.56, 95% CI: 0.41–0.78; OR separated or divorced vs married/common law: 0.54, 95% CI: 0.37–0.78).

## 4. Discussion

This study aimed to quantify the impact of the COVID-19 pandemic on the management of CP from the perspective of Canadians living with this condition. When just over a third of participants experienced changes in their pharmacological treatments, changes in physical and psychological approaches were the most important with 7 of 10 participants reporting such disruptions. Lack of access to clinics and exercise facilities was an important driver, and many participants needed to compensate for having to stop their usual treatments. Various sociodemographic and clinical factors were associated with the likelihood of experiencing changes in pain treatments during the pandemic.

In response to the COVID-19 pandemic, clinical practice recommendations have been rapidly issued by expert panels in the field of CP management.^16,19,57^ However, the research community was urged to produce epidemiological data that could help characterize the impact of the pandemic among individuals living with CP and inform interventions to reduce its effects.^14^ Data collection that can inform risk and/or management of drug shortages and assessment of the impact of the COVID-19 crisis on health care utilization and outcomes for non–COVID-19 diseases were identified as research priorities.^55^ To the best of our knowledge, the present Pan-Canadian study is the first of its kind to quantify the impact of the pandemic on the pharmacological and physical/psychological treatments of CP.

### 4.1. Prevalence and reasons for changes in pain treatments

Disruptions in physical/psychological management approaches to CP were the most prevalent (68%). The fact that fewer participants were impacted in terms of their pharmacological treatments (38% reported changes during the pandemic and less than half of them reported COVID-related reasons for such changes) suggests that relatively effective measures were put in place for many patients (eg, deliveries from pharmacies, telemedicine, prescription prolongation, pharmacist extensions of controlled drug prescriptions, etc.). It should also be pointed out that 35.7% of them did not need to renew pain medication since the beginning of the pandemic. Surprisingly, drug shortages and uncertainties around the use of NSAIDs, which had been widely broadcast throughout the media, were reported only by a minority of participants (3.3% and 3.2%). Changes to pharmacological treatment approaches were nonetheless reported (eg, 8.7% reported increase of medication intake to compensate for stopping physical/psychological treatments because of the pandemic), and further research is needed to investigate this issue to identify other strategies that could be implemented in future instances of health crisis. Health care professionals could also draw on the existing literature about optimal pharmacological treatments after natural disasters to identify new avenues^45,49^

As for physical/psychological treatments, the impact of the pandemic is deplorable because those approaches are often hard to implement and maintain despite their importance for pain management.^3,12^ Both our descriptive and multivariable results converge regarding the presence of an association between changes in pharmacological and physical/psychological treatments, which underline the importance of adopting the multimodal treatment paradigm^10^ in all interventions. As a lack of access to health care services and exercise facilities was the most common reason for changes in physical/psychological treatments during the pandemic, the development of interventions, policies, and knowledge transfer initiatives should target these problems. Practical recommendations for the rapid introduction of virtual interventions in response to the pandemic is already a hot topic in the field of pain management.^23^ Short videos suggesting alternatives when the usual physical/psychological treatments are not feasible could also be beneficial, and social media were suggested as useful dissemination tools in that regard.^18,46^

Incidental findings emerged from reading through the high number of verbatim answers. For example, some participants had to reintroduce or increase opioids despite tapering-off before the pandemic. Greater opioid requirements were indeed expected after exacerbation of pain symptoms in response to psychological stress brought by the pandemic^57^ and were reported in clinical settings.^33,40^ Other participants in our study reported using more cannabis products (medical or not) or even alcohol to ease their pain.

### 4.2. Changes in pain treatments and relations to clinical symptoms

Changes in pain condition since the beginning of the pandemic, pain intensity, and the presence of psychological distress symptoms were found to be significantly associated with change in pain treatments. Although the cross-sectional nature of the study affects the assessment of temporality between such variables, there are reasons to believe that changes in pharmacological and physical/psychological pain treatments resulted in symptoms degradation and not the opposite. First, because only a few participants (2.5%–11.6%) reported changes in their pain condition as the reason for pharmacological or physical/psychological treatment modification, we can presume that changes in pain treatments preceded modifications in pain symptoms. Second, even if both improvement and degradation of pain symptoms (as compared with unchanged symptoms) were associated with changes in pain treatments, only a minority of participants reported improvement of their pain condition in our study (5%). It is possible that the few participants who reported improvement of pain symptoms had success when switching to new treatment avenues (eg, positive lifestyle adjustments during confinement such as stretching and exercises at home every day with the family instead of attending weekly exercise classes).

### 4.3. Other factors associated with changes in pain treatments

Participants with generalized pain (for which the most likely reason is fibromyalgia^27^) were less likely to report changes in their pharmacological treatments. This association could be explain because their drug regimen may differ from that of patients with other types of pain conditions.^43,44,58^ In fact, trends in prescriptions dispensed before, during, and after the COVID-19 pandemic varied depending on the drug class^64^ and, as stated earlier, challenges surrounding access to some specific drug classes used in pain management arose during the pandemic. Participants who reported receiving pain care in a FMG were less likely to report changes in their pharmacological treatments than those treated in other clinical settings such as private clinics. Service continuity strategies used in such clinical settings should be further investigated. As expected, employment status changes during the pandemic and having needed to renew pain medication during this period were factors associated with changes in pharmacological pain treatments. Regardless of pain severity and all other variables included in our model, age and sex were both associated with the likelihood of reporting changes in physical/psychological pain treatments. This result could inform targeted prevention strategies of treatment disruptions and innovative treatment alternatives (eg, targeting females and young adults). Provincial differences were found in this study; as opposed to the province of Quebec, chances of reporting changes in pain treatments were increased in the provinces of British Columbia (as for pharmacological and physical/psychological treatments) and Alberta (as for physical/psychological pain treatments). Although Quebec was the most severely affected province in terms of the number of COVID-19 cases and deaths, all 3 provinces ranked in the top 4 of hot zones. Factors such as the variability in the public health responses of each province may explain this result.

### 4.4. SARS-CoV-2 and pain symptoms

The COVID-19 pandemic and associated public health measures have been identified as factors that could exacerbate symptoms of noninfected people living with CP (eg, lack of access to pain treatments and numerous/persistent stressors).^14^ One might also expect new onset of pain after SARS-CoV-2 infection and its complications (postviral syndrome),^11,14^ or even in healthy populations who experienced pandemic-related stress and risk factor exacerbation.^14^ At the preclinical level, some pain models suggested that SARS-CoV-2 infection could induce pain,^61^ whereas some data revealed possible pain relief.^48^ In our study, only 24 participants had been diagnosed with COVID-19, which limits our capacity to draw statistically valid conclusions about their experience, how the infection affected their pain, or the attention given to their pain when receiving COVID-related care. More large clinical studies are thus clearly needed.

### 4.5. Strengths and limitations

The core strengths of this study are its ability to capture data across multiple provinces in Canada and its capacity to do so during the peak of the first COVID-19 pandemic wave. Our study sample was comparable with previously described random surveys of individuals living with CP in terms of: (1) age (mean age: 49.7 vs 46.6–48.4),^6,47,62^ (2) proportion of workers (35% vs 38%–44%),^6,47^ (3) proportion of participants living with pain for more than 10 years (47.2% vs 46%–46.7%),^6,56^ and (4) pain intensity (mean 0–10 score: 6.1 ± 1.8 vs 6.3–6.9).^5,47^ The overrepresentation of women (83.5% vs 55%–65.3%^5,6,47,62^) is, however, a limitation of this study. This may be partly because of our web-based sampling method that favoured female participation as they are known to be more inclined to use Facebook^13^ or work in an online environment.^42^ Nevertheless, having achieved the recruited of a substantial number of men (n = 427) allowed us to stratify our main measures according to sex and adjust for sex in our multivariable models. Therefore, we believe that the participation bias was minimized.

It should be noted that the assessment of the reasons why and how participants modified their treatments was not standardized (open-ended questions). For the identification of variables associated with changes in pain treatments during the pandemic, the possibility of type II error was reduced because of the substantial sample sizes used in the multivariate models (n = 2135 and 1903). Nevertheless, our data did not permit the assessment of associations between changes in pain treatments and ethnic minority subgroups (too few representatives), income categories, or gender constructs (variables not prioritized in comparison with COVID-related items for the benefit of a shorter questionnaire), although such subgroup analyses were suggested relevant in the context of the COVID-19 pandemic after our study started.^4,25,66^ Overall, there is reason to believe that pain management challenges identified in this study may represent the situation of many patients living in countries where similar public health measures were applied. However, since the impacts seem to vary depending on participants' province of residence, the exact figures quantifying these impacts should be generalized with caution.

## 5. Conclusion

Our study highlights the significant impact the COVID-19 pandemic had, and probably continues having, on access to CP relief. The quantification of such an impact is essential to inform and prioritize interventions in prevision of future waves of the pandemic or ulterior similar disasters. A priority would be, of course, to maintain continuity of care for individuals suffering from CP despite the pandemic. Health care professionals and support groups organizations should implement virtual care options to supplement safe in-person care. Online interventions and knowledge transfer activities could also prioritize informing and empowering people living with CP regarding alternative physical/psychological approaches when the usual ones are not feasible. Let us not forget that the non-COVID challenges of CP management are still present^10^ and have been amplified by the COVID-19 health crisis. Harnessing the web could enhance treatment access for people living with CP well beyond the COVID-19 pandemic.

## Disclosures

The authors have no conflicts of interest to declare.
